# Involvement of impaired CD8^+^ mucosal-associated invariant T cells and myeloid-derived suppressor cells in polycystic ovary syndrome

**DOI:** 10.1186/s12958-021-00861-7

**Published:** 2021-11-30

**Authors:** Mengting Zhu, Yuping Xu, Caihua Li, Zhimin Lu, Kaihuan Bi, Kangxia Wang, Peipei Guo, Huanhuan Jiang, Yunxia Cao

**Affiliations:** 1 Reproductive Medicine Center, Department of Obstetrics and Gynecology, the First Affiliated Hospital of Anhui Medical University, No 218 Jixi Road, Hefei, 230022 Anhui China; 2 NHC Key Laboratory of study on abnormal gametes and reproductive tract (Anhui Medical University), No 81 Meishan Road, Hefei, 230032 Anhui China; 3 Key Laboratory of Population Health Across Life Cycle (Anhui Medical University), Ministry of Education of the People’s Republic of China, No 81 Meishan Road, Hefei, 230032 Anhui China; 4 Anhui Province Key Laboratory of Reproductive Health and Genetics, No 81 Meishan Road, Hefei, 230032 Anhui China; 5 Biopreservation and Artificial Organs, Anhui Provincial Engineering Research Center, Anhui Medical University, No 81 Meishan Road, Hefei, 230032 Anhui China; 6 Reproductive Medicine Center, Department of Obstetrics and Gynecology, the First Affiliated Hospital of Anhui Medical University, Wanshui Road Nr.120, Hefei, 230000 China

**Keywords:** PCOS, Immune, MAIT cells, MDSCs, Metabolic dysfunction

## Abstract

**Background:**

Immune dysfunction is one of the mechanisms to promote polycystic ovary syndrome (PCOS). Various immune cells have been reported to be involved in the development of PCOS. Meanwhile, the disturbance of metabolism is closely related to PCOS. The aim of this study is to explore the association of mucosal-associated invariant T (MAIT) cells and myeloid-derived suppressor cells (MDSCs) with the metabolic dysfunction in PCOS.

**Methods:**

68 PCOS patients and 40 controls were recruited in this study and we collected the peripheral blood of participants’ during their follicular phase. The frequencies of MAIT cells and MDSCs were determined by flow cytometry after being stained with different monoclonal antibodies. And the concentrations of cytokines were determined by ELISA.

**Results:**

Compared to controls with normal metabolism, the frequency of MDSCs, CD8^+^MAIT cells and CD38^+^CD8^+^MAIT cells were significantly decreased in PCOS patients with normal metabolism, however, proportion of CD4^+^MAIT cells exhibited a noticeable increase. Similar results of CD8^+^MAIT, CD38^+^CD8^+^MAIT cells and reduced expression of IL-17 were observed in PCOS patients with metabolic dysfunction as compared to controls with metabolic disorders. PCOS patients with excessive testosterone levels displayed significantly decreased levels of CD8^+^MAIT, CD38^+^CD8^+^MAIT cells, MDSCs and Mo-MDSCs as compared to PCOS patients with normal testosterone concentrations. PCOS patients with abnormal weight showed a lower level and activation of CD8^+^MAIT cells. On the contrary, they displayed an enrichment of CD4^+^MAIT cells. PCOS patients with glucose metabolic disorder displayed a remarkable dysregulation of MDSCs and Mo-MDSCs. MDSCs were positively correlated with MAIT cells. Negative correlations between the frequency of CD8^+^MAIT cells, CD38^+^CD8^+^MAIT cells and body mass index were revealed. CD4^+^MAIT cells positively correlated with BMI. Mo-MDSCs were found to be negatively related to the levels of 2hour plasma glucose and HOMA-IR index.

**Conclusion:**

The impairment of CD8+MAIT cells and MDSCs is involved in the metabolic dysfunction of PCOS.

## Introduction

Polycystic ovary syndrome (PCOS), is one of the most prevalent endocrine diseases during the reproductive age in female [1]. Approximately 8-13% women are affected by PCOS worldwide [2]. Patients with PCOS usually show typical clinical symptoms, including menstrual irregularity (oligomenorrhea or amenorrhea), hirsutism, acne and infertility. Nowadays, the metabolic dysfunction in PCOS has been payed close attention by physicians and researchers, because PCOS patients are frequently diagnosed with diabetes/insulin resistance, obesity and other long-term health problems [3–5]. The causes of PCOS have been studied for decades, however, the pathogenesis of PCOS still remains unclear. Recently, researches have focused on the association between immune disorders and PCOS, such as dysfunction of macrophages, dendritic cells and T regulatory cells (Tregs) [6–11].

MAIT (Mucosal-associated invariant T) cells, one kind of unconventional T lymphocytes, which are characterized by a semi-invariant T cell receptor (TCR). A restricted α chain (Vα7.2-Jα33 in humans and Vα19-Jα33 in mice) and one of the several β chains make up the TCR, which has been revolutionarily conserved [12–14]. In human, MAIT cells have been defined as CD3^+^CD161^+^Vα7.2^+^cells. According to expression of CD4 and CD8, MAIT cells group into three groups: CD8^+^MAIT cells and CD4^−^/CD8^−^(double negative, DN) MAIT cells and CD4^+^MAIT cells [15]. It has been reported that MAIT cells are relatively rich in peripheral blood (PB), liver, and other tissues [16]. Besides, recent studies have demonstrated that MAIT cells were involved in tumor, autoimmune diseases and other diseases [17–19].

Alteration of MAIT cells’ frequency and function in metabolic disfunction, such as diabetes/insulin resistance and obesity, have been demonstrated. Carolan et al. showed a dysfunction in MAIT cells in circulation of obesity adults [20]. Magalhaes and his colleagues reported abnormalities of circulating MAIT cells from type 2 diabetes mellitus(T2D) and obese patients [21, 22]. These results revealed a close association between MAIT cells and disorders of metabolism. PCOS is closely related to metabolic irregularity, we hypothesize that MAIT cells are related to PCOS.

MDSCs (Myeloid-derived suppressor cells) have also been considered to have a vital role in the immunopathogenesis in many types of diseases [23, 24]. MDSCs represent an intrinsic part of the myeloid-cell lineage and are revealed to have a strong immunosuppressive function. There are two subsets of MDSCs: monocytic MDSC (Mo-MDSC), and polymorphonuclear MDSC (PMN-MDSC) [25]. Published reports have demonstrated that MAIT cells have a potency of inducing MDSCs [26]. At present, there are no studies that explore the role of MAIT cells as well as MDSCs and the association between them in PCOS. The purpose of this study was to investigate the relationship between MAIT cells and MDSCs with metabolic dysfunction in PCOS patients.

## Materials and methods

### Patient selection

In this study, we recruited 68 patients diagnosed with PCOS and 40 age-matched women without PCOS as the controls from July 2019 to October 2021 in the First Affiliated Hospital of Anhui Medical University. The diagnostic criteria of PCOS were chosen according to the Rotterdam European Society for Human Reproduction. The BMI of all PCOS patients were more than 18kg/m^2^, including normal weight and obese patients. The controls consisted of healthy women and non-PCOS women but with metabolic dysfunction. All participants without pharmacological intervention including all kinds of hormone medicines, immunotherapies and other medicines (for example, Metformin, Atorvastatin) in six months. PCOS patients who had autoimmune disorder were also excluded. Extra exclusion criteria include (1) age>40 years or age<20 years; (2) suffering from acute infection or a state of inflammation; (3) other endocrine diseases, such as Cushing’s Syndrome and thyroid diseases. All participants volunteered to enroll in this study and signed a written consent inform. The process was approved by The Ethics Review Board of the First Affiliated Hospital of Anhui Medical University (No. PJ2020-12-38). Clinical and biochemical characteristics of all participants were summarized in Table 1.

**Table 1:** Characteristics of PCOS patients vs controls

	PCOS patients	Controls	*P*-value
Age, (year)	28.10±9.11	29.25±4.11	0.9897
BMI, (kg/m^2^)	26.26±4.29	24.93±4.15	0.1207
FSH, (IU/L)	6.46±1.70	7.08±1.90	0.0848
LH, (IU/L)	12.43±9.60	6.37±2.10	0.0002
LH/FSH	2.28±3.04	0.94±0.38	0.0073
E2, (pmol/L)	139.1±76.27	154.4±65.23	0.2907
T (nmol/L)	6.95±14.42	1.26±0.54	0.0142
PRL, (ng/ml)	22.31±49.28	16.41±8.36	0.4552
FPG, (mmol/L)	5.62±1.23	5.39±0.56	0.2716
2h PG, (mmol/L)	6.92±2.00	6.80±0.64	0.7088
FINS, (pmol/L)	16.70±14.0	12.75±6.54	0.0975
2h INS, (pmol/L)	52.40±40.50	39.55±17.92	0.0607
HOMA-IR	4.35±3.81	3.10±1.77	0.0547

This table represented the clinical and biochemical characteristics of PCOS patients (*n* =68) and controls (*n* =40). All values were expressed as mean±SD. *P*-values were calculated by using an unpaired Student's t-test. *BMI* body mass index, *FSH* follicular stimulating hormone, *LH* luteinizing hormone, *E2* estradiol, *T* testosterone, *PRL* prolactin, *FPG* fasting plasma glucose, *2h PG* 2hour plasma glucose, *FINS* fasting insulin, *2h INS* 2hour insulin, *HOMA-IR* homeostasis model assessment of insulin resistance

### Sample collection

We collected peripheral blood from each participant in the morning during their menstrual day 2-3. According to the Biocoll (Biochrom, Berlin, Germany) protocol, we obtained the peripheral blood mononuclear cells with density gradient centrifugation. We additionally collected plasma. The peripheral blood mononuclear cells and plasma were cryopreserved in the cryopreservation medium at-80°C.

### Flow cytometry analysis

Cells were treated with FcR Blocking Reagent (Biolegend, Germany) before being stained with human anti-bodies. MAIT cells and MDSCs were stained with conjugated antibodies. The conjugated antibodies and cell staining procedure were same with our previous study [27]. According to manufacturer's instructions, compensation control and cell acquisition was performed by FACSVerse and FACSuite software. Flow cytometry results were analyzed by FlowJo software (Tree Star, Ashland, OR).

### ELISA assay in serum

According to the manufacturer's protocol and procedure in our previous study, TGF, IL-6,10,12, 18, 17, and IFN-γ from plasma were analyzed by ELISA kit (Multisciences Biotech, Hangzhou, China) [27].

### Statistical analysis

We analyzed statistics by using GraphPad Prism software (GraphPad Software, San Diego, CA, USA). Statistical analyses of the differences between means in two groups were performed using unpaired, two-tailed t-test. We performed Spearman analysis to find the correlation. Correlation coefficients were presented as r. *P*-value <0.05 was considered significantly different.

## Results

### Positive correlation of MAIT cells and MDSCs in PB of PCOS patients

**Fig. 1:**
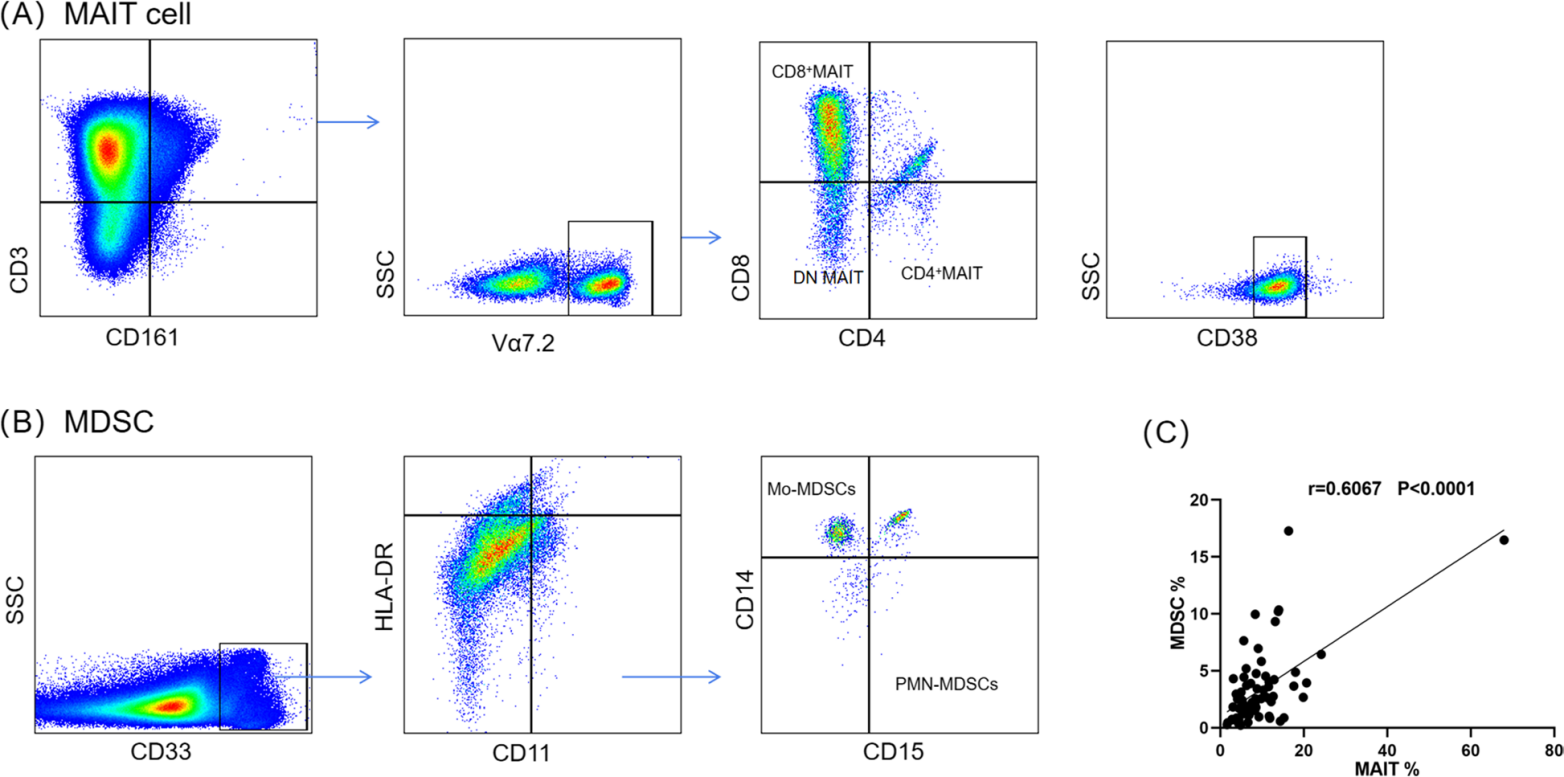
Analysis of MAIT cells and MDSCs in peripheral blood from patients by flow cytometry. Representative pseudocolor with the gating strategy identifying CD8^+^, CD4^+^, DN MAIT (A). CD3^+^CD161^+^ Vα7.2^+^ cells were gated at first. Basing on CD4 and CD8, and three subsets were identified: CD8^+^, CD4^+^ and DN MAIT cells. At last, cells which had expression of CD38 (CD38^+^) were selected. The gating strategy identifying MDSCs (B). Firstly, CD33^+^ cells were gated and then HLA-DR^-/low^ and CD11b^+^ cells were isolated. Next, based on CD14 and CD15, the cells were divided into Mo-MDSC (CD14^+^CD15^-^) and PMN-MDSC (CD14^-^CD15^+^). MAIT cells were positively correlated with MDSCs (C). MAIT cell, mucosal-associated invariant T cell; MDSC, myeloid-derived suppressor cell; DN, double negative; Mo-MDSC, monocytic myeloid-derived suppressor cell; PMN-MDSC, polymorphonuclear myeloid-derived suppressor cell

Figure 1 showed the gating strategy of MAIT cells (Fig. 1A) and MDSCs (Fig. 1B) in peripheral blood. MAIT cells were divided into DN, CD8^+^ and CD4^+^ three subpopulations. Finally, CD38^+^ cells were isolated. CD38 was considered as a marker of MAIT cells activation [28]. MDSCs were divided into two groups via flow cytometry, consisting of Mo-MDSCs and PMN-MDSCs. A positive correlation was clarified between the percentage of MAIT cells and MDSCs (Fig. 1C, *r* = 0.6067, *P* < 0.0001).

### Alteration of MDSCs, MAIT cells in PCOS patients as compared with controls

**Fig. 2:**
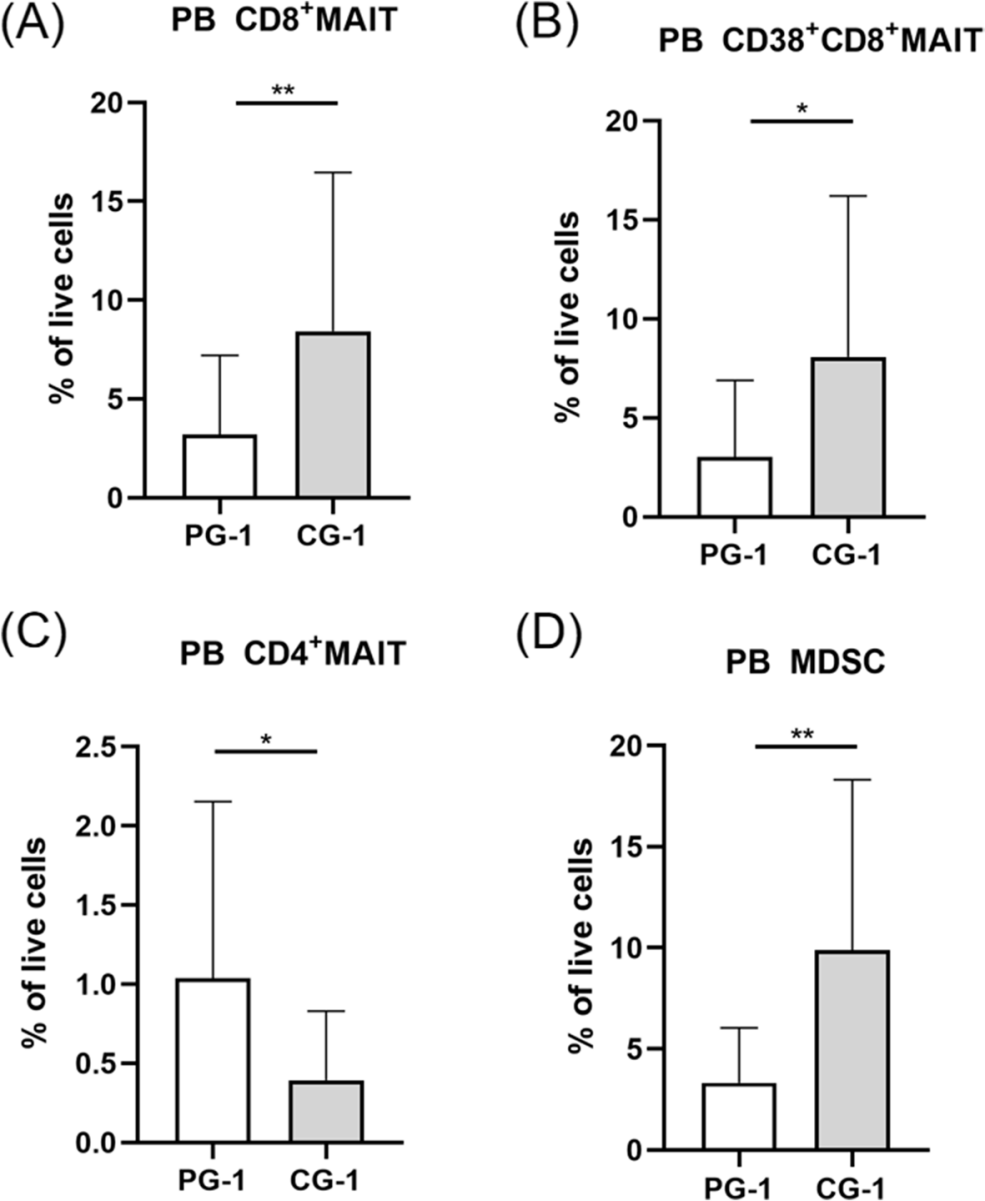
Percentages of MAIT cells subsets and MDSCs in circulation of PCOS patients without metabolic dysfunction (PG-1) and controls without metabolic disorders (CG-1). (A) displayed lower expression of CD8^+^MAIT cells inPG-1. PG-1exhibited a remarkable down-regulation of CD38 on CD8^+^ MAIT cells than CG-1(B). CD4^+^MAIT cells showed an enhancement in PB of PG-1(C). Expression of MDSCs significantly reduced in PG-1than CG-1(D). *P < 0.05, **P < 0.01. PG-1, PCOS group 1; CG-1, control group 1. MAIT cell, mucosal-associated invariant T cell; PB, peripheral blood; MDSC, myeloid-derived suppressor cell

**Fig. 3:**
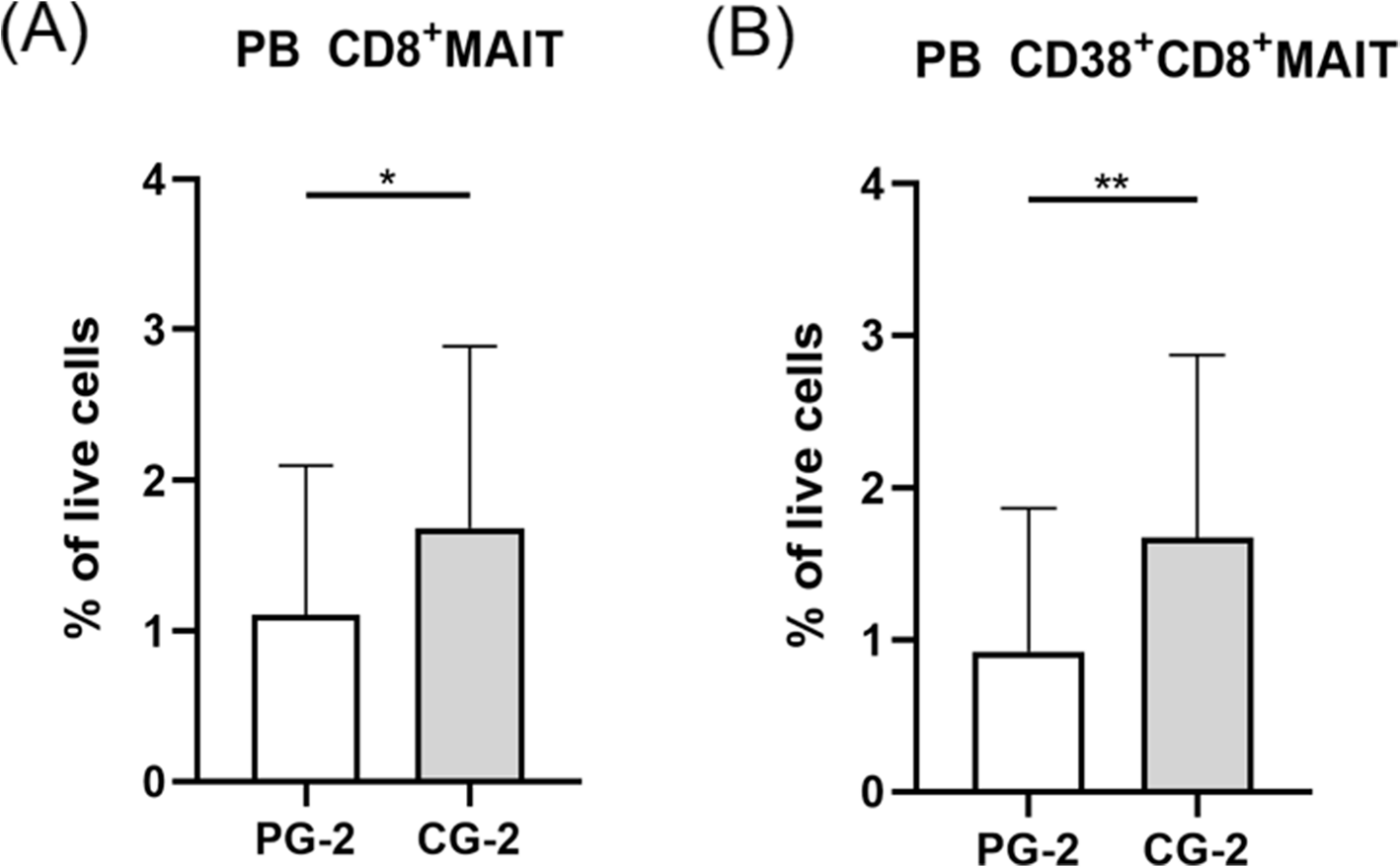
Analysis of MAIT cells in PCOS patients with metabolic dysfunction. Decreased frequencies of CD8^+^MAIT and CD38^+^CD8^+^MAIT cells were revealed in PG-2 (A, B). *P <0 .05, **P < 0.01. MAIT cell, mucosal-associated invariant T cell; PB, peripheral blood. PG-2, PCOS group 2; CG-2, control group 2

To demonstrate the involvement of MAIT cells and MDSCs in PCOS, both PCOS patients and controls were divided into different groups. Patients without metabolic dysfunction were divided into PCOS group 1 (PG-1) and PCOS patients with metabolism disturbance were divided into PCOS group 2 (PG-2). Meanwhile, controls with normal metabolism were grouped into control group 1 (CG-1) and controls accompanied by metabolic disorders were grouped into control group 2 (CG-2). Comparing withCG-1, PCOS patients with normal metabolism showed a significant decrease of CD8^+^ MAIT cells frequencies (Fig. 2A, *P* <0.01). The expression of CD38 in CD8^+^MAIT cells was prominently decreased in PCOS patients with normal metabolism (Fig. 2B, *P* <0.05). However, there was an accumulation of CD4^+^MAIT cells (Fig. 2C, *P* < 0.05). The percentages of MDSCs were distinctly decreased of PCOS patients with normal metabolism (Fig. 2D, *P* < 0.01). Meanwhile, PG-2 displayed decreased frequencies of CD8^+^ and CD38^+^CD8^+^MAIT cells in comparison with CG-2 (Fig 3A-B). Furthermore, concentration of IL-17 in PG-2 was significantly lower than CG-2 (Table 2). The summarized results of all cytokines were displayed in the Tables 2 and 3.

**Table 2:** cytokines in plasma of PG-2 and CG-2

Cytokines (pg/ml)	PG-2	CG-2	*P*-value
IL-6	3.02±3.82	1.30±1.42	0.0561
IL-10	8.09±8.15	6.88±5.99	0.5536
IL-12	1.52±2.19	1.44±0.75	0.8726
IL-17	2.69±3.97	7.11±8.56	0.0058
IL-18	120.6±89.61	84.71±72.12	0.1199
IFN-γ	5.97±5.24	6.21±6.10	0.8722
TGF	3955±1161	3727±886.3	0.4384

This table displayed the cytokines results of PG-2 (*n* =45) and CG-2 (*n* =20). Participants in PG-2 were PCOS patients with metabolic dysfunction. All controls in CG-2 were non-PCOS women with metabolic dysfunction. All values were expressed as mean±SD. *P*-values were calculated by using an unpaired Student's t-test. *TGF* transforming growth factor, *IL* Interleukin, *IFN-γ* Interferon-γ, *PG-2* PCOS group 2, *CG-2* control group 2

**Table 3:** cytokines in plasma of PG-1 and CG-1

Cytokines (pg/ml)	PG-1	CG-1	*P*-value
IL-6	1.60±1.56	1.37±1.94	0.6684
IL-10	9.39±15.07	6.07±6.54	0.3671
IL-12	0.81±0.23	0.95±0.33	0.1167
IL-17	4.85±15.93	10.25±38.62	0.5429
IL-18	119.5±76.75	104.3±71.92	0.5074
IFN-γ	5.82±7.31	6.42±8.54	0.8072
TGF	4008±1567	3988±1456	0.9658

This table displayed the cytokines results of PG-1 (*n* =23) and CG-1 (*n* =20). Participants in PG-1 were PCOS patients without metabolic disorders. All controls in CG-1 were healthy reproductive women. All values were expressed as mean±SD. *P*-values were calculated by using an unpaired Student's t-test. *TGF* transforming growth factor, *IL* Interleukin, *IFN-γ* Interferon-γ, *PG-1* PCOS group 1, *CG-1* control group 1

### The attenuation of MAIT cells subpopulation and MDSCs of PCOS patients with excessive testosterone levels

**Fig. 4:**
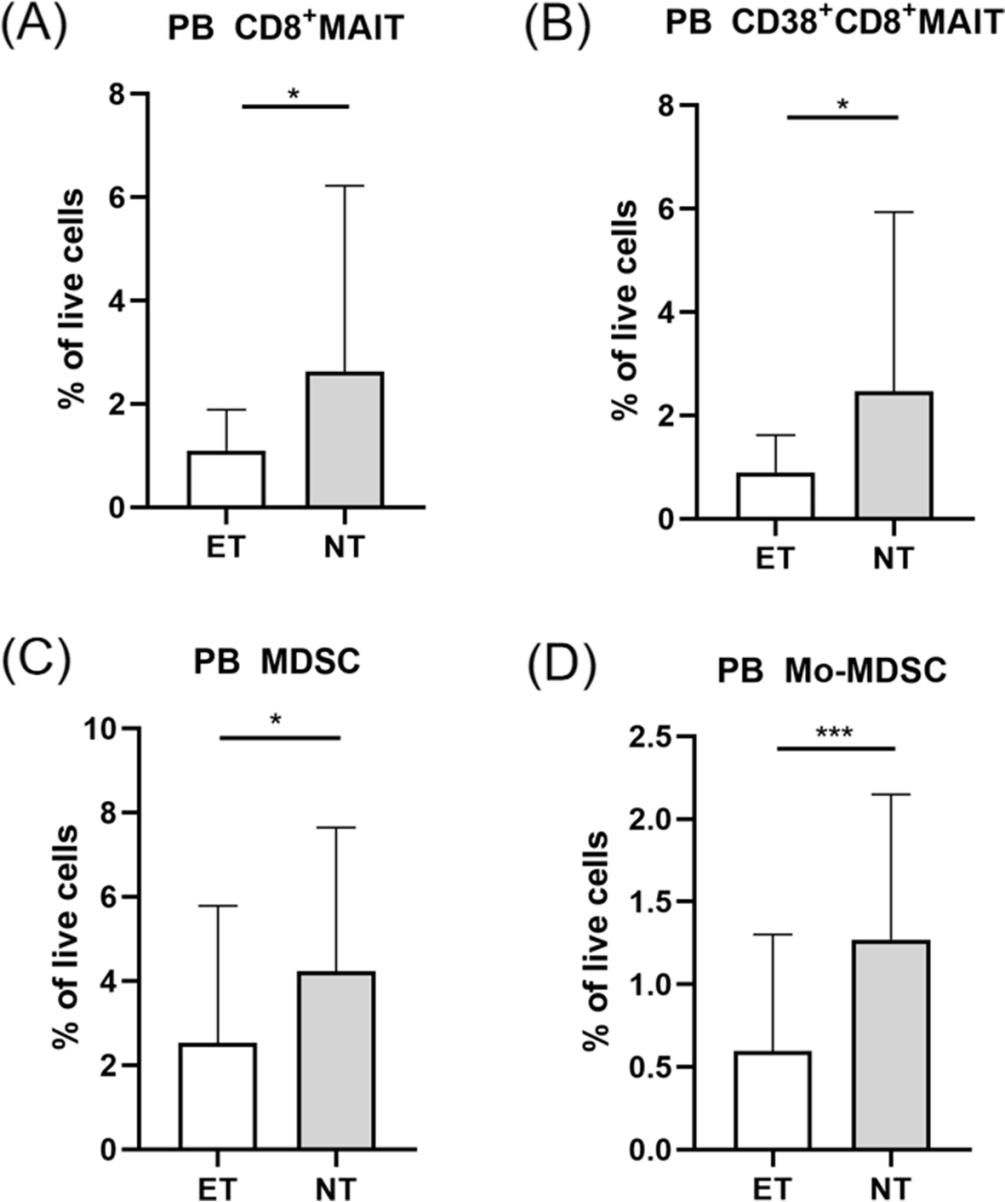
Distribution of circulatory MAIT cells subpopulations and MDSCs of PCOS patients with excessive testosterone levels and normal testosterone levels. PCOS patients with excessive testosterone had significantly decreased levels of CD8^+^MAIT and CD38^+^CD8^+^MAIT cells (A, B). Compared to NT group, MDSCs and Mo-MDSCs frequency of ET group were significantly decreased (C, D). *P <0 .05, ***P < 0.001. MAIT cell, mucosal-associated invariant T cell; MDSC, myeloid-derived suppressor cell; Mo-MDSC, monocytic myeloid-derived suppressor cell; PB, peripheral blood; NT, normal testosterone; ET, excessive testosterone

Based on the testosterone reference range of reproductive women in our hospital (0.2-2.0 nmol/L), all PCOS patients were grouped into two subgroups, excessive testosterone (ET) subgroup and normal testosterone (NT) subgroup. Patients in ET subgroup revealed lower distribution of CD8^+^MAIT cells (Fig. 4A) and CD38^+^CD8^+^MAIT cells (Fig. 4B). Meanwhile, PCOS patients with excessive testosterone levels showed obvious reduction of MDSCs (Fig. 4C) and Mo-MDSCs (Fig. 4D).

### The levels of MAIT cells of PCOS patients with abnormal weight and correlations between these cells and BMI

**Fig. 5:**
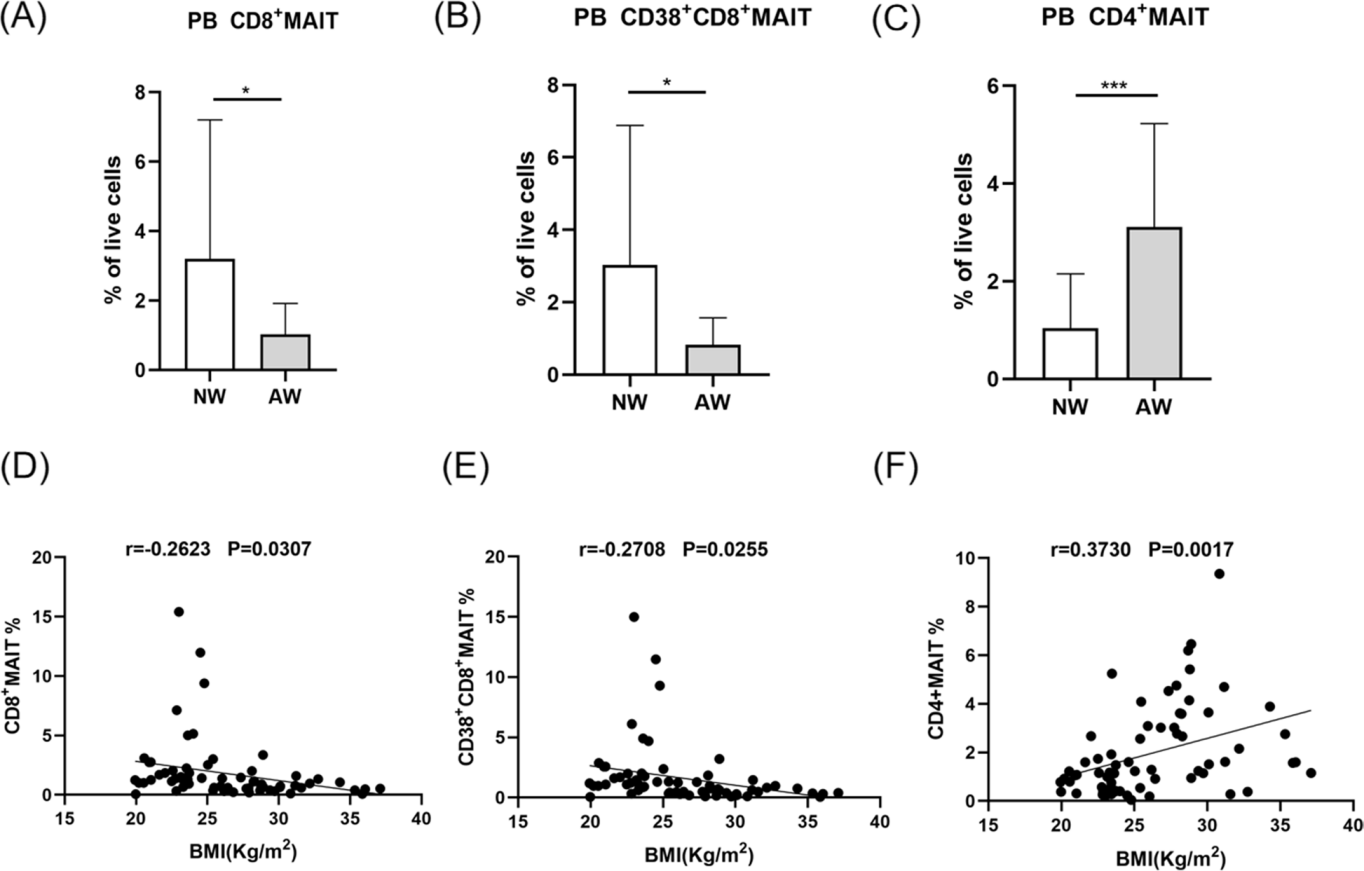
Histogram represents frequency of MAIT cells subsets in NW and AW cohorts. CD8^+^MAIT cells and CD38^+^CD8^+^ MAIT cells showed higher expression in the NW group (A, B). Patients of AW group showed a dramatic elevation of CD4^+^MAIT cells (C). The negative correlation between the frequency of CD8^+^MAIT cells as well as CD38^+^CD8^+^ MAIT cells and BMI (D, E). A positive association between the CD4^+^MAIT cells and BMI was discovered (F). *P <0 .05, ***P < 0.001. MAIT cell, mucosal-associated invariant T cell; PB, peripheral blood; NW, normal weight; AG, abnormal weight; BMI, body mass index

There were 45 patients without glucose metabolic dysfunction in PCOS group. According to BMI, these patients were divided into two subgroups, the normal weight subgroup (NW) (18< BMI≤24) and the abnormal weight subgroup (AW) (the BMI>24) There were 23 patients in NW group and 22 patients in AW group. Peripheral blood CD8^+^MAIT cells (Fig. 5A, *P* < 0.05) and CD38^+^CD8^+^MAIT cells (Fig. 5B, *P* < 0.05) both significantly reduced in AW group compared with those from NW group. While CD4^+^MAIT cells obviously displayed an enhancement in AW group (Fig. 5C, *P* < 0.001). The frequency of CD8^+^MAIT cells (Fig. 5D, *r* =-0.2623, *P*=0.0307) and CD38^+^CD8^+^MAIT cells (Fig. 5E, *r* =-0.2708, *P=*0.0255) negatively correlated with BMI. The frequency of CD4^+^MAIT cells positively correlated with BMI (Fig. 5F, *r* =0.3730, *P* =0.0017).

### Attenuation of MDSCs from PCOS patients with glucose metabolic disorder

**Fig. 6:**
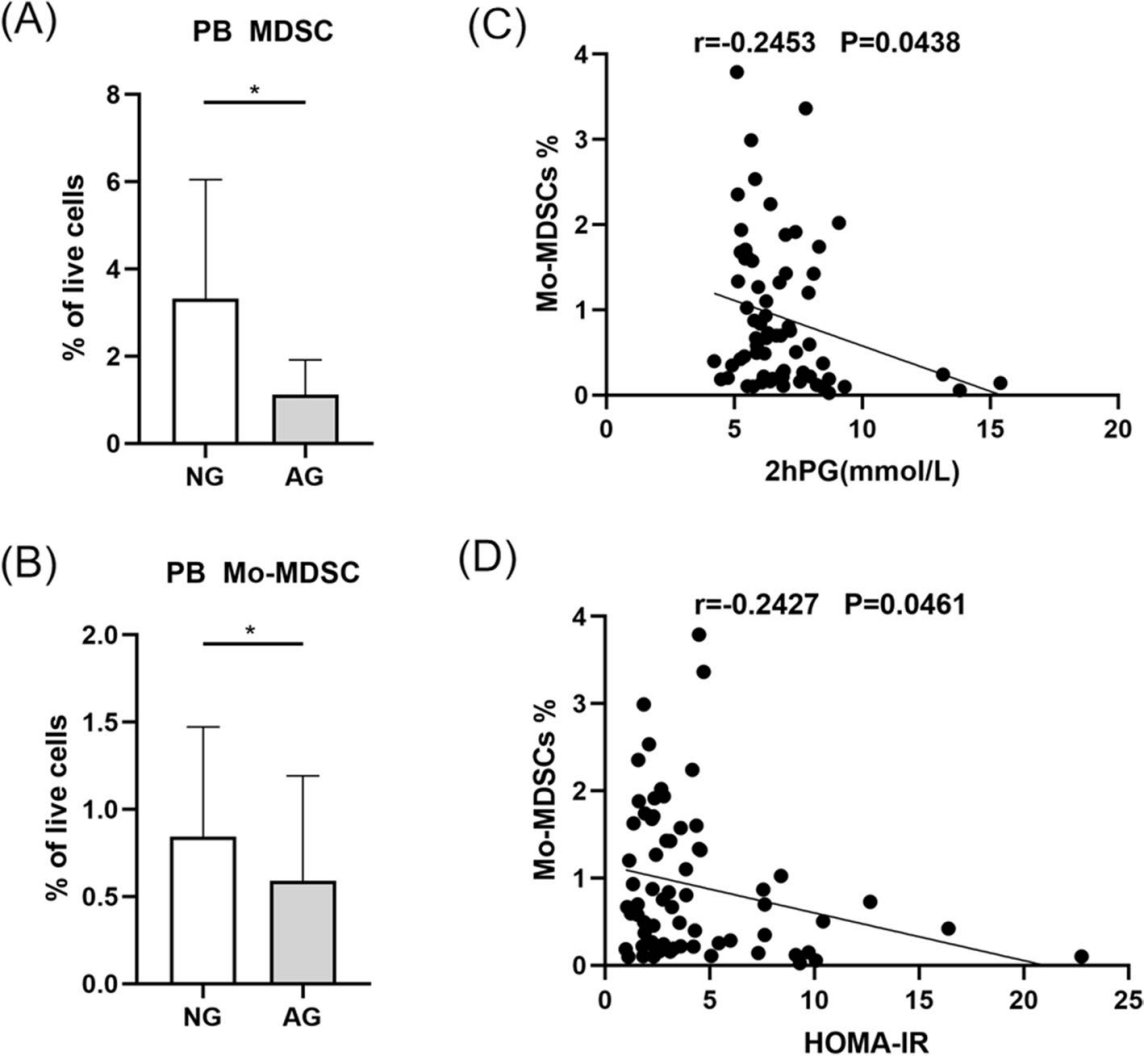
Histogram showing MDSCs and Mo-MDSC frequency in two groups, including NG group and AG group. Compared to NG group, MDSCs frequency of AG group were significantly decreased (A). The percentage of Mo-MDSCs decreased significantly in AG group (B). C-D displayed the negative associations between Mo-MDSCs and the level of 2hour PG and HOMA-IR index of PCOS patients. *P <0.05. MDSC, myeloid-derived suppressor cell; Mo-MDSC, monocytic myeloid-derived suppressor cell; PB, peripheral blood; NG, normal glucose; AG, abnormal glucose; 2h PG, 2hour plasma glucose; HOMA-IR, homeostasis model assessment of insulin resistance

32 patients with normal weight were divided into two groups, normal glucose (NG) subgroup and abnormal glucose (AG) subgroup. PCOS patients with a fasting glucose level less than 6.1mmol/L and a 2hour glucose level less than 7.8mmol/L were assigned to the NG group. The remaining patients were divided into the AG group. There were 23patients in the NG group and 9 patients in the AG group. Compared with NG group, AG group have decreased levels of MDSC (Fig. 6A, *P* < 0.05). And AG group have lower levels of Mo-MDSC, as compared to that from NG group (Fig. 6B, *P* < 0.05). Negative correlation between Mo-MDSC and the level of 2hour plasma glucose (Fig. 6C, *r* =-0.2453, *P* = 0.0438) as well as HOMA-IR index (Fig. 6D, *r* =-0.2427, *P* = 0.0461) were also found. For the frequencies of MAIT cells and PMN-MDSCs population, no significant differences between subgroups were found.

## Discussion

In this study, we intended to evaluate the potential correlation of MAIT cells subpopulations and MDSCs with metabolic disorders in PCOS for the first time. Lower expression of CD8^+^MAIT cells, CD38^+^CD8^+^MAIT cells and MDSCs were displayed in ET subgroup among PCOS patients, in addition to this, both PG-1 and PG-2 showed decreased levels of these cells. These reductions indicated that the disorders of MAIT cells and MDSCs seem to be associated with PCOS. And the distributions of circulatory CD8^+^MAIT cells and Mo-MDSCs were negatively correlated with metabolic dysfunction in PCOS. All these findings suggested that the altered distribution of CD8^+^MAIT cells and MDSCs may be involved in development of PCOS via influencing the metabolism. It has been reported that the abnormality of Th17 cells percentage was associated with PCOS [29]. Due to the same secretion of IL-17, MAIT cells have been reported to have a similar function of Th17 cells [30]. In this study, a decreased frequency of CD8^+^MAIT cells in PCOS patients was revealed. Moreover, CD38^+^CD8^+^MAIT cells, known as activated MAIT cells, were also reduced. Except attenuation of CD38^+^CD8^+^MAIT cells, the concentration of Il-17 decreased to varying degrees in both PG-1 and PG-2 groups. Our results indicated that the impaired frequency and function of CD8^+^MAIT cells might have a close link with PCOS. Early studies have suggested that CD8^+^MAIT cells played a protective role in some diseases [31, 32]. Consistent with previous studies, our study also revealed the protective role of CD8^+^MAIT cells in PCOS. Nonetheless, CD4^+^MAIT cells displayed an expansion in PCOS. It has been known that the functional roles and frequencies of MAIT cells differ between different subsets and different diseases [33, 34]. In this study, our results also support the distinct roles of CD8^+^MAIT and CD4^+^MAIT cells. Recently, PCOS was also recognized as a chronic inflammatory disease [35, 36]. Therefore, our findings would suggest that the protective role of CD8^+^MAIT cells in PCOS are weakened, which is similar to the results from other chronic inflammatory diseases.

One of the major problems from patients with PCOS is weight concern . In our study, we found that with the increase of BMI, the frequencies of CD8^+^MAIT cells and CD38^+^CD8^+^MAIT cells decreased. Whereas, the proportion of CD4+MAIT cells displayed increased levels. Previous researches have depicted that obese adults displayed a depletion of circulating MAIT cells [20, 21]. Touch et al. have revealed that obese human showed an impaired frequency of MAIT cells [37]. A recent study investigated by Li et al. has suggested a considerable decrease of MAIT cells frequency and a distinct dysfunctional state of MAIT cells in obese patients [38]. CD8+MAIT cells were the majority of MAIT cells, we implied that the lipid metabolic disorder was mainly affected by the decreased amount and weakened function of CD8^+^ MAIT cells in PCOS patients [16]. We indicated that the reduction of CD8+MAIT cells might promote the development of obesity in PCOS patients.

MDSCs have been indicated to have a close relation with MAIT cells in various pathological processes [28]. Haeryfar et al. suggested that MAIT cells could induce MDSCs [26]. Nevertheless, a direct connection between MAIT cells and MDSCs has not been identified in PCOS. In our study, we found a significant reduction of MDSCs and a positive relationship between MAIT cells and MDSCs in the PB of PCOS patients. One explanation would be that lessened MAIT cells have an impaired function of induction of MDSCs. Though there are no studies about the role of MDSCs in PCOS, another group of immunosuppressive cells, Tregs, has been indicated to be reduced in PCOS patients [11, 39]. Tregs play an important role in maintaining the balance of immune system by suppressing effector T cells. Reduced immunosuppression is a key factor in the overreaction in inflammatory response. Based on the similar immunosuppressive properties of MDSCs and Tregs, the two populations are expected to share a similar role in different diseases [40, 41]. Therefore, we suggested that an impaired immunosuppressive microenvironment with reduced MDSCs might be a cause of long-term chronic inflammation in PCOS.

Furthermore, our findings also revealed that PCOS patients with disorders of glucose metabolism had a strong reduction of MDSCs and Mo-MDSCs. What’s more, with the progression of glucose metabolic dysfunction, the frequencies of MDSCs and Mo-MDSCs decreased. The roles of MDSCs in metabolic diseases have been widely studied. Yin et al. have demonstrated that transferred MDSCs could down-regulate autoimmune responses and prevent diabetes onset in mice model [42]. Researchers have previously clarified that MDSCs could improve metabolic dysfunctions and act as a protector in obesity [43]. Combined with these results, our study indicated that a decreased frequency of MDSCs and Mo-MDSCs might sharpen the metabolic dysfunction in PCOS patients.

## Conclusion

The outcomes of our research gave a clue that the combined reduction of CD8^+^MAIT cells and MDSCs, and increased population of CD4^+^MAIT cells might contribute to the metabolic disorders in PCOS. These findings enriched our comprehension of the metabolic dysfunction in PCOS and provided a new point about the pathogenesis of PCOS. . However, the limitation of this study is that we only analyzed the PB from PCOS patients, which might not present the immune status in ovary microenvironment. These observations support the hypothesis that impaired CD8^+^ MAIT cells and MDSCs are involved in PCOS at the cellular level. Further in-depth researches of these immune cells in PCOS will be carried out in the future.

## Data Availability

The datasets analyzed during the current study are available from the corresponding author on reasonable request.

## Abbreviations

PCOS: Polycystic ovary syndrome
Tregs: T regulatory cells
MAIT cell: Mucosal-associated invariant T cell
TCR: T cell receptor
DN: Double negative
PB: Peripheral blood
T2D: Type 2 diabetes mellitus
MDSC: Myeloid-derived suppressor cell
Mo-MDSC: Monocytic myeloid-derived suppressor cell
PMN-MDSC: Polymorphonuclear myeloid-derived suppressor cell
TGF: Transforming growth factor
IL: Interleukin
IFN-γ: Interferon-γ
PG-1: PCOS group 1
PG-2: PCOS group 2
CG-1: Control group 1
CG-2: Control group 2
ET: Excessive testosterone
NT: Normal testosterone
BMI: Body mass index
NW: Normal weight
AG: Abnormal weight
NG: Normal glucose
AG: Abnormal glucose
HOMA-IR: Homeostasis model assessment of insulin resistance
